# Genetic and clinical analysis in Chinese patients with retinitis pigmentosa caused by *EYS* mutations

**DOI:** 10.1002/mgg3.1117

**Published:** 2020-01-15

**Authors:** Yan Sun, Jian‐kang Li, Wei He, Zhuo‐shi Wang, Jin‐yue Bai, Ling Xu, Bo Xing, Jian‐guo Zhang, Lusheng Wang, Wei Li, Fang Chen

**Affiliations:** ^1^ Shenyang He Eye Specialist Hospital Shenyang China; ^2^ He University Shenyang China; ^3^ Department of Computer Science City University of Hong Kong Kowloon Hong Kong; ^4^ BGI‐Shenzhen Shenzhen China; ^5^ School of Basic Medicine Qingdao University Qingdao China; ^6^ BGI Education Center University of Chinese Academy of Sciences Shenzhen China; ^7^ Laboratory of Genomics and Molecular Biomedicine Department of Biology University of Copenhagen Copenhagen Denmark

**Keywords:** blue blindness, mutation spectrum, panel‐based targeted exome sequencing, retinitis pigmentosa

## Abstract

**Background:**

Panel‐based targeted exome sequencing was applied to identify the pathogenic variants and genetic characteristics of retinitis pigmentosa (RP) in two Chinese families, and to gain a deeper understanding of the relationship between clinical manifestations and genotypes.

**Methods:**

A total of 17 subjects, comprising two probands (total patients: four subjects) and their family member, were recruited in this study. All subjects underwent comprehensive ophthalmic examinations and clinical evaluations, and the complete history and medical records were collected according to the standard procedures. All participants were screened using the multigene panel test (Target_Eye_792_V2 chip), and Sanger sequencing was used to confirm the candidate variants.

**Results:**

Among these two families, a total of three novel mutations in the *EYS* gene were identified in patients, including a homozygous frameshift mutation c.9252_9253insT detected in two patients in one family, and the compound heterozygous splicesite mutation c.5644+2T>C and frameshift mutation c.1920_1923delTGAG detected in two patients in the another family. All patients in both families had early onset of night blindness and poor visual acuity, and with typical posterior capsule opacification. The mutation co‐segregated within all recruited individuals. In addition, one patient with compound heterozygous mutations was found to have typical blue‐blindness symptoms and detected a previously reported disease‐causing mutation c.235G>A in *OPN1SW* gene, which caused blue blindness manifestations and was first discovered in patient combined with RP causative genes.

**Conclusions:**

Panel‐based targeted exome sequencing was used to identify three novel variants of RP causative gene, and we also detected a known pathogenic variants of blue‐blindness causative genes in two patients. Our finding will provide a powerful basis for genetic counseling and enhance our current understanding of the genetics factors for RP families.

## Graphical abstract

We applied panel‐based targeted exon sequencing to explore the pathogenic variation spectrum and genetic characteristics of retinitis pigmentosa populations in two Chinese families, and have a deeper understanding of the relationship between clinical manifestations and genotypes.

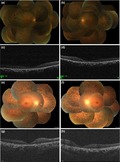

## 1 INTRODUCTION

Hereditary retinal degeneration is an important neurodegenerative disease and the leading cause of genetic hereditary blindness in the world. According to different clinical symptoms, hereditary retinal degeneration can be divided into: macular degeneration (MD), retinitis pigmentosa (RP), and cone‐rod dystrophy etc., and the common feature of these diseases is that the structure or function of the retina is disordered and gradually leads to the death of photoreceptor cells (Berger, Kloeckener‐Gruissem, & Neidhardt, 2010; Wright, Chakarova, Abd El‐Aziz, & Bhattacharya, 2010). At present, about 200 retinal degeneration genes have been identified, but the specific molecular mechanism of retinal degeneration caused by mutations in these genes is not comprehensive enough (Goldberg, Moritz, & Williams, 2016; Hoon, Okawa, Della Santina, & Wong, 2014).

RP is the most common form of retinal degeneration, is one of the most common cause of blindness with a habitual manifestation of loss of function in photoreceptor cells and retinal pigment epithelium in significant clinical and hereditary heterogeneity. The typical early lesions of RP mainly start from the peripheral retina and gradually develop into the fovea of the macula. The main symptoms include night blindness and progressive reduction of visual field, and ultimately developed into tubular vision and blindness (Hartong, Berson, & Dryja, 2006). There were approximately 1.5 million RP patients around the world, and there is a significant difference in accidence between different region and different ethnic groups. According to statistics, the current incidence of RP in the world is 1/3,000–1/7,000, and the incidence in China is about 1/3,500–1/5,000 (Hartong et al., 2006). The average age of onset of these patients is generally between 20 and 64 years old, and the severity of the illness and the specific time of occurrence vary from person to person (Bertelsen, Jensen, Bregnhøj, & Rosenberg, 2014; Hu, 1982; Sohocki et al., 2001).

The genetic forms of RP mainly include autosomal dominant RP (adRP), autosomal recessive RP (arRP), X‐linked RP (XL‐RP), and maternal inheritance (mitochondrial inheritance) and digenic inheritance. So far, about 100 RP causative genes have been identified, including 56 non‐syndromic forms of disease‐causing genes, 12 USH‐syndromic disease‐causing genes, and 17 BBS‐syndromic disease‐causing genes (Daiger, Sullivan, & Bowne, 2013; Huang et al., 2015). Huang et al. (2015) determined the pathogenic variations of 55% patients with RP by molecular diagnosis (Huang et al., 2015). A total of 1,243 patients with RP were enrolled in a large Chinese cohort study, the result showed that 72.08% of patients received a molecular diagnosis, and the top 17 genes account for 75.63% of the diagnosed cases. Among these top 17 genes, the *EYS* gene accounts for 7% of the diagnosed cases. A total of 76 genes were identified in this cohort, and the blue‐blindness *OPN1SW *genes were not detected in this study (Gao, Li, et al., 2019). So far, the *EYS* gene mutations have been detected and verified in the 5%–16% autosomal recessive RP families, and the commonest types of mutations were frameshift and missense mutations (Barragán et al., 2010; Abd El‐Aziz et al., 2010; Littink et al., 2010). There were few studies and reports on *EYS* genes in China, and 69 disease‐causing mutations of *EYS* gene have been reported, which are insufficient for clinical phenotypic description (Xiaoqiang, Yingjie, & Shaowan, 2019).

In this study, we performed a comprehensive clinical and molecular assay of two RP families caused by mutations in the *EYS* gene. Our results not only provide accurate and reliable diagnosis, but also expand the existing mutation spectrum and provide a reference for panel‐based genetic diagnosis design.

## 2 METHODS

### 2.1 Subjects and ethics statement

A total of 17 subjects from two families (total patients: four subjects) were recruited in Shenyang He Eye Specialist Hospital from June 2017 to January 2019. The study was approved by the Ethics committee of the He Eye Specialist Hospital of He university (approval number: IRB [2016] K001.01). All participants or their guardians signed written informed consent in accordance with the Helsinki Declaration. Among these two families, the first family (No. ARRP‐01) consist of two generations and six members were recruited, including two affected individuals (II‐1 and II‐2) diagnosed with RP and four unaffected individuals; the second family (No. ARRP‐02) consist of three generations and 11 members were recruited, including two affected individuals (II‐1 and II‐3) diagnosed with RP and nine unaffected individuals. A total of seven individuals in two families performed targeted exome sequencing.

### 2.2 Clinical assessments

All participants with confirmed *EYS* pathogenic mutations underwent comprehensive ophthalmic examinations, including best‐corrected visual acuity, slit‐lamp biomicroscopy, noncontact intraocular pressure, color vision, wide‐field fundus imaging (Optos PLC), macular spectral‐domain optical coherence tomography (SD‐OCT, Spectralis HRA + OCT, Heidelberg Engineering, Inc), full‐field electroretinography, and fundus autofluorescence (Spectralis HRA COCT). Genomic DNA was extracted from peripheral blood using the FlexiGene DNA Kit (Qiagen) according to the manufacturer's protocol.

### 2.3 Panel‐based targeted exome sequencing analysis

We designed a panel‐based high‐throughput targeted enrichment method to capture exon regions of 792 genes associated with common inherited eye diseases (Table S1). The Capture Panel (Target_Eye_792_V2 chip) was custom designed and produced by the Beijing Genomics Institute (BGI). On average, the mean coverage depth was more than 300X for genome DNA, and the coverage of targeted region is close to 99.9% using BGISEQ 2000 platform (BGI, Inc.).

We aligned sequence reads to the reference human genome (UCSC hg 38) using the Burrows–Wheeler aligner version 0.7.10 (BWA‐MEM). Then, the sequence data obtained were analyzed as described elsewhere (Gao, Li, et al., 2019; Gao, Qi, et al., 2019; Hu et al., 2019; Li et al., 2019). Previous reported variants were determined using Human Gene Mutation Database (HGMD, http://www.hgmd.cf.ac.uk/ac/index.php), ClinVar (https://www.ncbi.nlm.nih.gov/clinvar/), and locus‐specific databases. Variants were classified as pathogenic, likely pathogenic, and novel variants of uncertain clinical significance according to the American College of Medical Genetics (Johnston & Biesecker, 2013; Stenson, Ball, Mort, Phillips, & Cooper, 2012). The obtained candidate variants were first verified by Sanger sequencing or quantitative real‐time polymerase chain reaction, then reviewed by clinical geneticists and ophthalmologists, and validation of variant segregates with the disease within the two families.

## 3 RESULTS

### 3.1 Cohort characteristics

**Figure 1:**
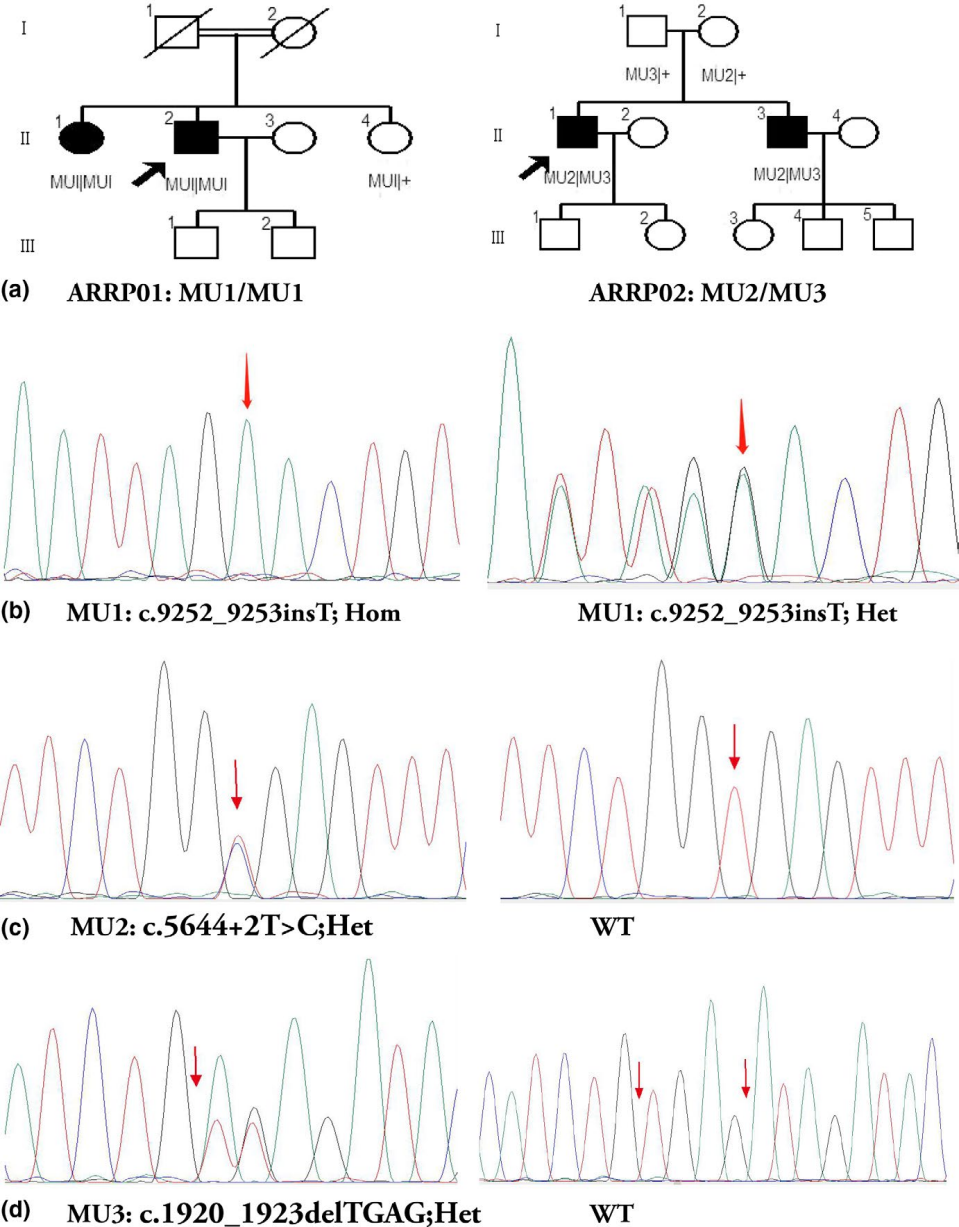
Pedigrees of two families with autosomal recessive retinitis pigmentosa (ARRP) and pathogenic variations were identified by Sanger sequencing in participants. (a) Pedigrees of two families. Squares represent males and circles represent females; black and white shades represent affected and unaffected individuals, respectively. Black lines indicate deceased individuals, and the probands were marked with an arrow. (b) Sanger sequencing of mutation No.1 (MU1): c.9252_9253insT, Hom and c.9252_9253insT, Het. (c) Sanger sequencing of mutation No.2 (MU2): c.5644+2T>C, Het and wild type. (d) Sanger sequencing of mutation No.3 (MU3): c.1920_1923delTGAG, Het and wild type

A total of 17 individuals from two families were recruited in this study (Figure 1a). The proband (II‐2) of the family ARRP‐01 was a 53‐year‐old male who developed symptoms of night blindness in both eyes at the age of 28, and his parents were consanguineous marriage and the mother denied pregnancy infection. As the disease progresses, the symptoms of night blindness begin to accelerate at the age of 50, and the binocular visual acuity was FC/FC (figure count). The proband (II‐1) of the non‐consanguineous family ARRP‐02 was a 37‐year‐old male who developed symptoms of mild myopia (−2.0D logMAR units) combined with night blindness in both eye at the age of 14. At the age of 23, he realized that the symptoms of night blindness began to accelerate. The family denied the consanguineous marriage, the infection during pregnancy, and the systemic examination showed no abnormalities. The visual acuity of the right eye was corrected to 0.1 logMAR units using a −4.0D spherical lens, and the visual acuity of the left eye was HM/0.1 M and could not be corrected in ARRP‐02:II‐1. In addition, using the standard Color Visual Checklist and the Farnsworth D‐15 Color Vision Test, the proband (ARRP‐02:II‐1) was diagnosed with rare blue‐blindness symptoms (TRITAN) (Figure S1d).

**Figure 2:**
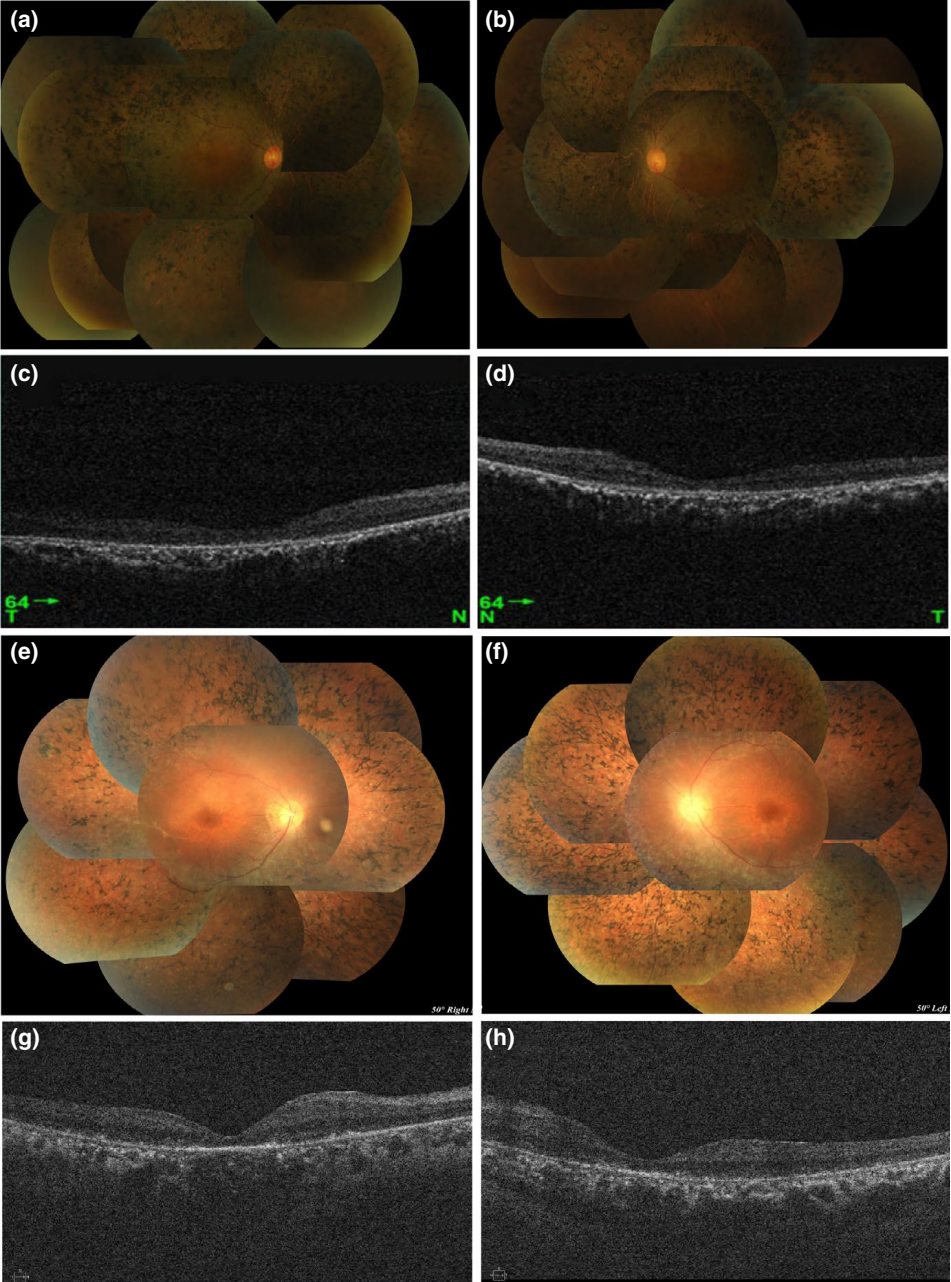
Color fundus puzzle and spectral‐domain optical coherence tomography (SD‐OCT) of macular regions of two probands. (a, b, e, and f) Color fundus puzzle of the proband II‐2 (family No.1: ARRP‐01) and the proband II‐1 (family No.2: ARRP‐02) bilaterally show the typical symptoms of RP, characterized by optic disc waxy pallor, attenuated retinal vessels, and the retina is atrophied and the color is blue‐gray. (c, d, g, and h) SD‐OCT of the macular of the probands II‐2 (ARRP‐01) and II‐1 (ARRP‐02) show the degenerative changes of retinal layers in both eyes, revealing the structural damages of both inner segment ellipsoid band and photoreceptor outer segment

All four patients showed typical symptoms of RP, the clinical manifestations included night blindness and progressive visual field stenosis, and all patients had a progressive bilateral decrease in visual acuity. Among these four patients, the average of onset of night blindness was 21 ± 7 (range, 14–28; median, 22). The fundus puzzle presents symptoms of typical bone spicule‐shaped pigment deposits distributed in the equatorial and peripheral regions of the retina, with typical complications including posterior cystic cataract. The visual field examinations of the patients of the two families have been unable to cooperate, but the dynamic field of view is <10°. Color fundus puzzle of the probands (ARRP‐01:II‐2 and ARRP‐02:II‐1) showed the typical symptoms of RP, characterized by optic disc waxy pallor, attenuated retinal vessels, and the retina is atrophied and the color is blue‐gray. In addition to the macular area, there is a large amount of typical bone spicule‐shaped pigment deposits distributed in the equatorial and peripheral regions of the retina (Figure 2a,b,e,f). SD‐OCT of the macular of the probands (ARRP‐01:II‐2 and ARRP‐02:II‐1) show the degenerative changes of retinal layers in both eyes, revealing the structural damages of both inner segment ellipsoid band and photoreceptor outer segment (Figure 2c,d,g,h). In the macular of both eyes, moderate retinal thinning along with structural changes in both inner segment ellipsoid band and photoreceptor outer segment was observed by means of SD‐OCT. The difference is that SD‐OCT examination shows that the proband ARRP‐02:II‐1 still retains the thinned ellipsoidal band (IS/OS) layer in the macular area of the eyes, and the degree of the retina atrophy in the macula is lighter than that of the proband ARRP‐01:II‐2. At the same time, the fovea of the macula showed abnormal morphology, and the high‐reflection band of the nerve fiber layer was clearer than the proband ARRP‐01:II‐2.

### 3.2 Genetic finding

A total of seven individuals of the two families were screened by the multigene panel test (Target_Eye_792_V2 chip), and Sanger sequencing was used to confirm the candidate variants (Figure 1b–d). Panel‐based targeted exome sequencing was performed in four patients with non‐syndromic RP, and no disease‐causing mutations were detected in other genes except for the *EYS* gene. A total of three novel variants were detected in this two families, including a splicesite mutation c.5644+2T>C (Het), and two frameshift mutations c.1920_1923delTGAG (Het) and c.9252_9253insT (Hom) were identified in four patients. All of the above three mutations cause the amino acid sequence of the encoded protein to change from the mutation locus, further impairing the function of the encoded protein. Using the UCSC Genome Browser (http://genome.ucsc.edu/cgi-bin/hgGateway), to perform sequence conservation analysis of mutant amino acids, we found that these mutations are highly conserved among primates (Figure S1a–c). Meanwhile, the above three variations were co‐segregated within all recruited individuals, and none of these three variants existed in any publicly available databases (Table 1). These data suggested that the *EYS* gene would be the most relevant disease‐causing gene in the four patients with diagnosed RP. Of these two families, the homozygous frameshift mutation c.9252_9253insT was detected in two patients (ARRP01:II‐1 and ARRP01:II‐2), and the compound heterozygous splicesite mutation c.5644+2T>C combined with frameshift mutation c.1920_1923delTGAG was detected in two other patients (ARRP‐02:II‐1 and ARRP‐02:II‐3), which were inherited from the father (c.1920_1923delTGAG) and the mother (c.5644+2T>C), respectively. In addition, one patient (ARRP‐02:II‐1) with compound heterozygous mutations were found to have typical blue‐blindness symptoms and detected a previously reported disease‐causing missense mutation c.235G>A in *OPN1SW *gene, which caused blue blindness manifestations and was first discovered in patient combined with retinitis pigmentosa (Table 1).

**Table 1:** Genetics finding in the two families with retinitis pigmentosa

Family ID	Gene	Mut name	Amino acid change	Exon intron ID	Zygous	Chr:por:mut	Functional change	G1000_AF	dbSNP_AF	ESP6500_AF	ExAC_AF	Clinical significance	Reference
ARRP‐01:II‐1	*EYS*	c.9252_9253insT	p.Ser3084Serfs9	EX44/CDS41	Hom	chr6:63720841:G>GA	Frameshift	0	0	0	0	P	Novel
ARRP‐01:II‐2	*EYS*	c.9252_9253insT	p.Ser3084Serfs9	EX44/CDS41	Hom	chr6:63720841:G>GA	Frameshift	0	0	0	0	P	Novel
ARRP‐01:II‐4	*EYS*	c.9252_9253insT	p.Ser3084Serfs9	EX44/CDS41	Het	chr6:63720841:G>GA	Frameshift	0	0	0	0	P	Novel
ARRP‐02:II‐1	*EYS*	c.5644+2T>C	—	Intron26	Het	chr6:64590221:A>G	SpliceDonor	0	0	0	0	P	Novel
	*EYS*	c.1920_1923delTGAG	p.Cys640Stopfs1	EX12/CDS9	Het	chr6:65295962:TCTCA>T	Frameshift	0	0	0	0	P	Novel
	*OPN1SW*	c.235G>A	p.Gly79Arg	EX1	Het	chr7:128775556:C>T	Missense	0.001	0.0003994	0.0002	0.0002553	P	Weitz et al. (1992)
ARRP‐02:I‐1	*EYS*	c.1920_1923delTGAG	p.Cys640Stopfs1	EX12/CDS9	Het	chr6:65295962:TCTCA>T	Frameshift	0	0	0	0	P	Novel
ARRP‐02:I‐2	*EYS*	c.5644+2T>C	—	Intron26	Het	chr6:64590221:A>G	SpliceDonor	0	0	0	0	P	Novel
ARRP‐02:II‐3	*EYS*	c.5644+2T>C	—	Intron26	Het	chr6:64590221:A>G	SpliceDonor	0	0	0	0	P	Novel
	*EYS*	c.1920_1923delTGAG	p.Cys640Stopfs1	EX12/CDS9	Het	chr6:65295962:TCTCA>T	Frameshift	0	0	0	0	P	Novel

Abbreviation: P, pathogenic; LP, likely pathogenic; VUS, uncertain clinical significance.

## 4 CONCLUSION

Abd El‐Aziz et al. (2008) first discovered the *EYS* (Eyes shut homolog) gene and found six different mutations in the *EYS* gene in patients with autosomal recessive RP. By genome analysis, Abd El‐Aziz et al. (2008) determined that the *EYS *gene contains 43 exons and spans 2.0 Mb, and was mapped to chromosome 6q12 (Abd El‐Aziz et al., 2008). Collin et al. (2008) performed genomic analysis of the RP25 locus by RT‐PCR and RACE, and identified a large transcript containing 10,475 nucleotides, including the 3‐prime untranslated region and poly‐A tail. The transcript encodes a protein comprising 3,165 amino acids and was predicted to contain a signal peptide secreted into the extracellular environment, 28 EGF‐like domains and 5 laminin A G‐like domains. BLAST analysis showed that the gene was the ortholog of the Drosophila “eyes closed” (eys) gene. RT‐PCR analysis of total RNA in various tissues of the human body indicates that the *EYS* gene is abundantly expressed in the retina (Collin et al., 2008). In the primate retina, the protein encoded by the *EYS* gene is not only highly aggregated near CC/TZ, but also was located in the outer segment of the photoreceptor and weakly expressed at the outer segment of the rod and at the end of the cone (Messchaert et al., 2018; Yu et al., 2016).

In the family ARRP‐01, a homozygous frameshift mutation c.9252_9253insT in the exon 44 of the *EYS* gene was detected, which resulted in a disorder in the downstream sequence of amino acid 3084 and resulted in a truncated termination. In the family ARRP‐02, a heterozygous mutation c.5644+2T>C in the intron 26 combined with heterozygous mutation c.1920_1923delTGAG in the exon 12 was detected. Among these two mutations, the splicing mutation c.5644+2T>C occurred in the intron, which may affect the splice of mRNA; the frameshift mutation c.1920_1923delTGAG will change the amino acid sequence of the encoded protein, and produced a truncated termination, further impairing the function of the encoded protein. Since the mRNA transcribed from the *EYS* gene containing these truncation mutations has a premature termination codons, the transcription product can be degraded by a nonsense‐mediated decay (NMD) mechanism, resulting in the loss of expression or loss of function of the *EYS* protein. Yu et al. (2016) also found that the C‐terminus of * EYS* is required for its functional expression in the retina, while nonsense, insertion, or deletion mutations in the *EYS* gene cause the mRNA of *EYS* gene to be degraded by a NMD mechanism (Yu et al., 2016).

According to the published data of *EYS* gene in Chinese cohorts, the age of onset of Chinese RP patients caused by *EYS* gene ranged from 8 to 45 years old, including 17.1% of the age of onset under 15 years old, 43.9% of the age of the onset ranged from 16 to 30, and the patients over 30 years old accounted for 39%. Among these two probands, the average age of onset was 28 years old (ARRP‐01:II‐2) and 14 years old (ARRP‐02:II‐1), both of them showed the symptoms of retina and choroidal atrophy. And except for the macular area, a large amount of typical bone spicule‐shaped pigment deposits distributed in the equatorial and peripheral regions of the retina. The difference is that SD‐OCT examination suggests that the proband ARRP‐02:II‐1 still retains the thinned ellipsoidal band (IS/OS) layer in the macular area, and the degree of retinal atrophy was slighter, and the high‐reflection band of the nerve fiber layer was clearer. We believe that differences in clinical symptoms of the fundus may be due to different types of mutations in the *EYS* gene. Kimiko et al. (2014) demonstrated that the type of mutation is related to the severity of RP symptoms. Patients with homozygous or compound heterozygous frameshift mutations have significantly decreased visual acuity, and the age of accelerated visual development is also advanced, while patients with single frameshift mutations have only visual acuity with slight decrease (Kimiko et al., 2014). Gu, Tian, Chen, and Zhao (2016) also confirmed that the clinical phenotype of homozygous frameshift mutations is more serious than homozygous missense mutations, so we speculate that the differences in fundus symptoms between the two probands may be caused by different mutation types (Gu et al., 2016).

Moreover, the proband (ARRP‐02:II‐1) was diagnosed with rare blue‐blindness symptoms (TRITAN) and detected a previously reported disease‐causing missense mutation c.235G>A (p.Gly79Arg/G79R) in *OPN1SW* gene. Blue‐blindness is caused by the mutation in the *OPN1SW* gene located on chromosome 7q32, which presented an autosomal dominant inheritance pattern. According to Weitz, Miyake, Shinzato, Montag, and Nathans (1992), blue‐blindness caused by G79R mutation in the *OPN1SW* gene has low penetrance through autosomal dominant inheritance, and Gly (positive charge residue) replaced by Arg (nonpolar residue) in the transmembrane domain may disrupt the folding, procession, or stability of the blue‐sensitive protein, resulting in loss of function of the protein (Littink et al., 2010; Sakmar, Franke, & Khorana, 1989; Weitz et al., 1992). Therefore, we speculate that the G79R mutation is the disease‐causing mutation of the proband ARRP‐02:II‐1, and this is the first report in patient combined with RP causative genes.

In summary, this study first discovered three novel pathogenic mutations in the *EYS* gene, expanded the pathogenic variation spectrum of the *EYS* gene, and studied the clinical effects of homozygous mutations and compound heterozygous mutations of the *EYS* gene on different patients.

## CONFLICT OF INTEREST

The authors declare that the research was conducted in the absence of any commercial or financial relationships that could be construed as a potential conflict of interest

## AUTHOR CONTRIBUTIONS

FC, WL, and L‐SW conceived and designed this study. YS, Z‐SW, LX, and WH recruited patients, performed clinical examinations, and interpretation. BX, J‐YB, and J‐GZ collected the clinical samples and clinical data. J‐KL and WL analyzed the sequencing data. WL and YS wrote and revised the manuscript.

[Correction added on 31 January 2020, after first online publication: In Author Contributions section, the author abbreviation ‘XS’ has been deleted.]

## Data Availability

The data that support the findings of this study have been deposited in the CNSA (https://db.cngb.org/cnsa/) of CNGBdb with accession code CNP CNP0000503.
