# Supplementary figures and images for: Genetic and clinical analysis in Chinese patients with retinitis pigmentosa caused by EYS mutations
Source: Mol Genet Genomic Med. 2020 Jan 15;8(3):e1117. doi: 10.1002/mgg3.1117 (PMC7057104; doi:10.1002/mgg3.1117)

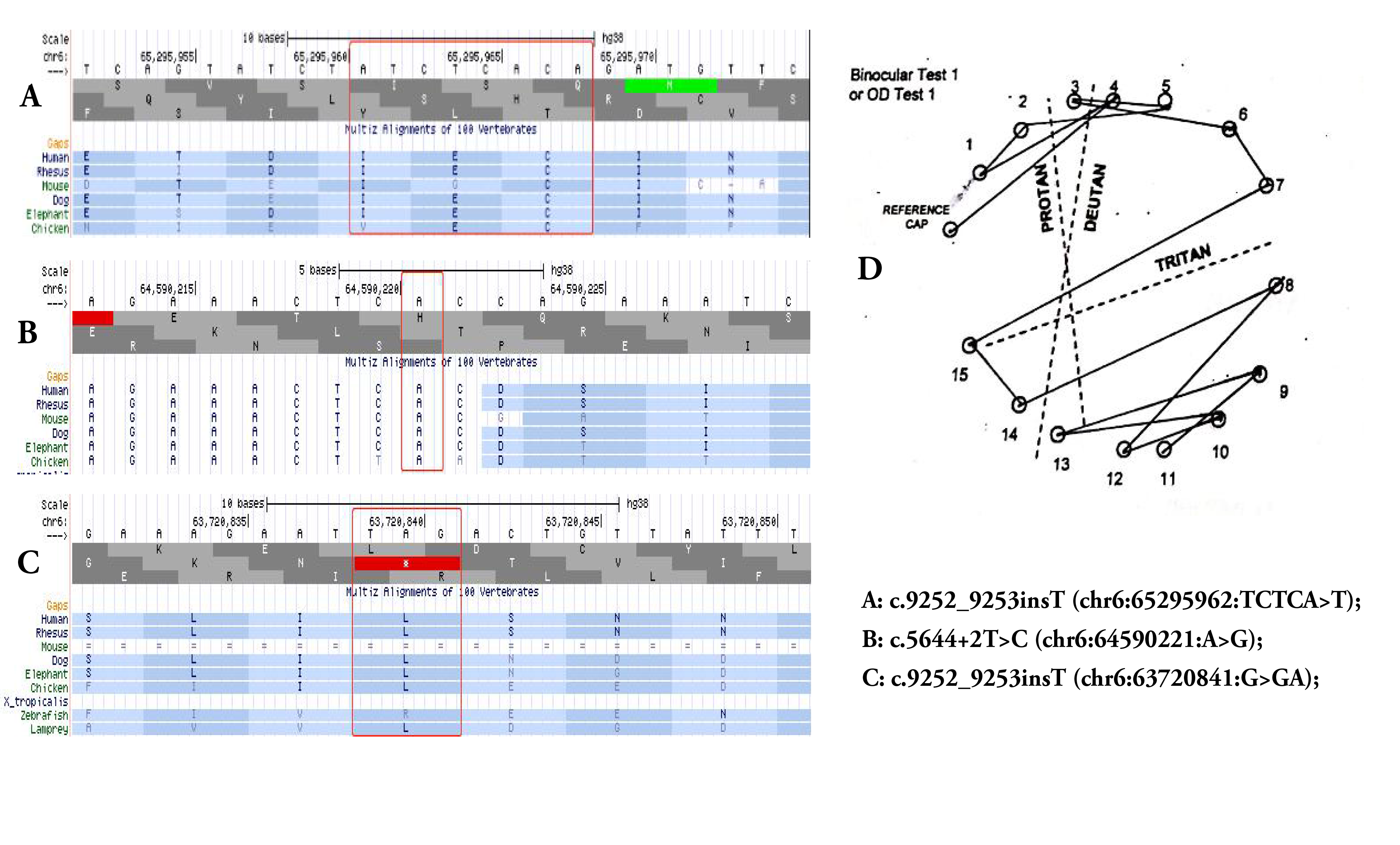

Supplement: Supplementary file 1 [file MGG3-8-e1117-s001.png]
